# Assessments of Heart Rate and Sympathetic and Parasympathetic Nervous Activities of Normal Mouse Fetuses at Different Stages of Fetal Development Using Fetal Electrocardiography

**DOI:** 10.3389/fphys.2021.652828

**Published:** 2021-04-08

**Authors:** Yoshiyuki Kasahara, Chihiro Yoshida, Masatoshi Saito, Yoshitaka Kimura

**Affiliations:** ^1^Department of Maternal and Fetal Therapeutics, Tohoku University Graduate School of Medicine, Sendai, Japan; ^2^Department of Maternal and Child Health Care Medical Science, Tohoku University Graduate School of Medicine, Sendai, Japan; ^3^Department of Obstetrics and Gynecology, Tohoku University Graduate School of Medicine, Sendai, Japan

**Keywords:** fetal ECG, autonomic nervous system, heart rate, fetal development, sympathetic nervous system, parasympathetic nervous system, heart rate variability

## Abstract

Heart rate is controlled by the activity of the autonomic nervous system: the sympathetic and parasympathetic nervous systems increase and suppress heart rate, respectively. To evaluate the activity of the autonomic nervous system, it is possible to determine heart rate variability using electrocardiography (ECG). During the fetal period, the heart and autonomic nerves develop in coordination; however, physiological changes, including autonomic nervous activities that occur during the fetal stage, remain largely unknown. Therefore, in this study, we measured ECG signals of mouse fetuses using our established method to evaluate the development of heart rate and autonomic nervous activity at different fetal developmental stages. We found that heart rate was significantly increased in fetal mice at embryonic day (E) 18.5 compared with that at E13.5, E15.5, and E17.5, indicating that fetal heart rate increases only at the stage immediately prior to birth. Interestingly, fetal parasympathetic nervous activity was reduced at E17.5 and E18.5 compared with that at E13.5, whereas fetal sympathetic nervous activity remained unchanged, at least from E13.5 to E18.5. These results indicate that parasympathetic activity rather than sympathetic activity affects fetal heart rate and that the decrease in parasympathetic activity toward the end of pregnancy could result in the observed increase in fetal heart rate.

## Introduction

Heart rate is largely regulated by the autonomic nervous system, which includes two anatomical divisions: the sympathetic and parasympathetic nervous systems (Wehrwein et al., 2016). The sympathetic nervous system increases heart rate, whereas the parasympathetic nervous system suppresses it. Heart rate variability (HRV) can be analyzed using electrocardiography (ECG) to assess the activity of the autonomic nervous system (Kimura et al., 1996).

In the fetal stage, the heart and autonomic nerves develop simultaneously; however, the physiological changes involving autonomic nervous activity that occur during the fetal stage have yet to be studied in detail. As described by Vegh et al. (2016), at embryonic day (E) 8.0, the developing heart can be recognized as a primary heart tube; at this early stage, the autonomic nervous system has yet to be developed. However, at E11.5, neural crest-derived cells (NCCs) delaminate from the neural tube and begin migrating ventrally and caudally. At approximately E12.5, the migrating NCCs begin to play a role in the development of cardiac innervation and conduction. For fetal mice, catecholamine expression is essential for embryonic cardiac development in both the preinnervation and postinnervation phases, and fetuses are completely dependent on noradrenalin from E9.5 to E13.5 (Vegh et al., 2016). Therefore, to study the coordinated development of the heart and autonomic nervous system in fetal mice based on physiological characteristics, it is necessary to study fetal development after E13.5.

Assessing the development of the autonomic nervous system during the fetal period is also important to elucidate disease mechanisms. According to the Developmental Origins of Health and Disease (DOHaD) concept, exposure to certain factors such as stress or infections during the prenatal period may cause long-term side effects. This paradigm provides an understanding of how the risk factors present during fetal development contribute to the occurrence of several diseases in later life. However, the relationship between fetal autonomic nervous system development and disease requires further attention, largely because it remains technically difficult to measure autonomic function in the fetal period.

Over the past decade, we have developed techniques for fetal ECG measurement in humans (Sato et al., 2011; Velayo et al., 2017; Oshio et al., 2018) and animals (Kimura et al., 1996; Minato et al., 2018; Kasahara et al., 2020); specifically, our techniques allow the evaluation of autonomic nervous activity using ECG collected from fetuses. We previously measured fetal ECG signals of fetal growth-restricted (FGR) mouse fetuses at E17.5 and found that short-term variability (STV), an indicator of parasympathetic activity, decreased (Minato et al., 2018). In another study, we measured fetal ECG signals of a mouse model of autism spectrum disorder (ASD), which we established by administering valproic acid to pregnant mice, and assessed the autonomic function of these mice at both E15.5 and E18.5 using power spectrum analysis; we found that sympathetic nerve function was attenuated during the fetal period (Kasahara et al., 2020). In general, power spectrum analysis of HRV provides information on the balance of the sympathetic and parasympathetic activities that occur during numerous physiological and pathophysiological conditions. The power spectrum of HRV can be divided into two main domains, i.e., low-frequency (LF) and high-frequency (HF) domains (Montano et al., 1994): LF represents the activities of sympathetic and parasympathetic nerves, HF represents the activity of parasympathetic nerves only, and the LF/HF ratio represents the activity of sympathetic nerves only.

Although it is possible to study the mechanisms of fetal pathophysiology by measuring fetal ECG, few studies have evaluated the activity of the normal autonomic nervous system in detail during each fetal developmental stage. Therefore, in the present study, we measured fetal ECG signals of mice at E13.5, E15.5, E17.5, and E18.5 to evaluate the development of the autonomic nervous system during the fetal period. Overall, our findings will help improve our understanding of the mechanisms underlying fetal HRV during fetal development.

## Materials and Methods

### Animals

All animal handling and experimental procedures were performed in accordance with the Guidelines for the Care of Laboratory Animals of Tohoku University Graduate School of Medicine and were approved by the Committee on Animal Experiments in Tohoku University, Sendai, Japan (study approval number: 2017MdA-334).

C57BL6/J mice (CLEA, Tokyo, Japan) were used in all experiments. C57BL6/J is the most widely used inbred strain of laboratory mouse; these mice have a homogenetic background. All mice were housed socially (3–5 mice in the same cage) in same-sex groups in a temperature-controlled environment under a 12-h light/12-h dark cycle (lights on at 08:00; lights off at 20:00), with food and water provided *ad libitum.*

### Fetal and Maternal ECG Measurements

We performed fetal and maternal ECG measurements according to methods described in our previous study (Kasahara et al., 2020). Female mice aged 7–19 weeks were mated with age-matched male mice in the evening and examined for the presence of a vaginal plug the next morning; we considered this day E0.5. Fetal and maternal ECG measurements were then performed on E13.5, E15.5, E17.5, and E18.5 to evaluate the physiological development of fetuses. Separate cohorts of experimentally naive pregnant female mice were used for each ECG experiment at E13.5, E15.5, E17.5, and E18.5.

The sample size of fetuses in this study was calculated based on our previous study (Kasahara et al., 2020). By calculating the sample size using the E18.5 results from this previous study, we found that the required sample size was 11 when the power was set to 0.80 (*β* = 0.2). In general, a sample size (*n*) of approximately 10 is appropriate for animal experiments; thus, based on the power calculation and the need to minimize the number of experiments for animal welfare reasons, we set the sample size in the present study to 10–12. We collected and analyzed fetal ECG data at E13.5 (*n* = 12 fetuses from 8 mothers), E15.5 (*n* = 10 fetuses from 5 mothers), E17.5 (*n* = 10 fetuses from 6 mothers), and E18.5 (*n* = 11 fetuses from 6 mothers).

To record fetal and maternal ECG, pregnant mice were anesthetized with subcutaneous administration of ketamine (Ketalar 500 mg, 100 mg/kg; Daiichi-Sankyo, Tokyo, Japan) and xylazine (Rompun 2% w/v solution, 10 mg/kg; Bayer, Leverkusen, Germany) and maintained under anesthetic with inhalational isoflurane (0.5%, 260 ml/min; Forane AbbVie Inc., Chicago, IL, United States). Anesthetic depth was assessed using a toe pinch test. Both ketamine (Behdad et al., 2013) and isoflurane (Khanjani et al., 2014) are sometimes administered to pregnant women during cesarean section without severe side effects. We used isoflurane in addition to ketamine–xylazine to reduce the amount of anesthetic required and to maintain stable anesthesia during fetal and maternal ECG measurements. In our previous studies, we confirmed that fetal ECG could be measured stably with this combination of anesthetics (Minato et al., 2018; Kasahara et al., 2020). The system for recording fetal ECG in embryonic mice was the same as that described in previous studies (Khandoker et al., 2018; Minato et al., 2018; Kasahara et al., 2020). Briefly, three needle electrodes were attached to the pregnant mother, and two needle electrodes were inserted into the uterus and attached to the chest and back of the fetus. Fetal ECG recordings were collected simultaneously from two randomly selected fetuses of a pregnant mouse. ECG signals (sampling frequency: 1000 Hz) of pregnant mice and their fetuses were recorded for 15 min using a biomedical amplifier and recording system (Polymate AP1532; TEAC, Tokyo, Japan).

### Data Analysis

The fetal and maternal ECG data processing and analysis methods used here followed those previously described by Kasahara et al. (2020). Briefly, from 15-min ECG recordings, 1 min of data between the 5th and 6th min was selected for analysis. However, if noise and/or temporary arrhythmia, tachycardia, or bradycardia was detected in the fetus during this period, 1 min of data was selected from between the 6th min and 10th min. Beat-to-beat intervals were determined from ECG data by calculating the time difference between two consecutive R-wave peaks (RR interval). The HRV of each fetus and mother was calculated from the RR interval of the fetal ECG. The power spectral density was calculated according to previous methods (Kimura et al., 1996; Kasahara et al., 2020) to assess the activities of autonomic nerves. From the power spectra, LF and HF areas were calculated using MATLAB software (version 2008b; MathWorks, United States). To simplify the analysis procedure, LF and HF data were transformed to their natural logarithms and defined as LF (ln) and HF (ln), respectively. We considered LF (ln) as an indicator of sympathetic and parasympathetic nerve activity, HF (ln) as an indicator of parasympathetic nerve activity, and the LF (ln)/HF (ln) ratio as an indicator of sympathetic nerve activity. We also analyzed the heart rate and STV, which is another indicator of parasympathetic nerve activity (van Ravenswaaij-Arts et al., 1993). In this study, data that were outside the ± 3 standard deviation range were excluded from further analysis. Average data were calculated and presented per fetus.

### Statistical Analysis

All data obtained from fetal and maternal ECG measurements were analyzed using SPSS (version 21; IBM, United States) and one-way analysis of variance (ANOVA) with a between-subject factor of developmental stage (gestational age; E13.5, E15.5, E17.5, and E18.5). Significant ANOVA results were further tested using Bonferroni *post hoc* comparisons. The alpha level was set at 0.05.

## Results

**FIGURE 1:**
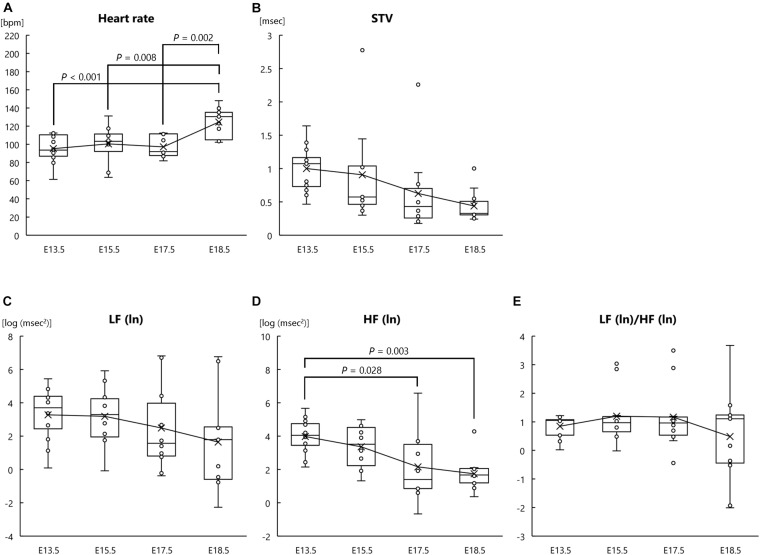
Heart rate and autonomic nervous activities at each fetal stage in normal mouse fetuses. **(A)** Heart rate, **(B)** short-term variability (STV), **(C)** natural logarithm of the low-frequency of the power spectrum [LF (ln)], **(D)** natural logarithm of the high-frequency of the power spectrum [HF (ln)], and **(E)** the LF (ln)/HF (ln) ratio at the following fetal stages: embryonic day (E) 13.5 (*n* = 12 fetuses from 8 mothers), E15.5 (*n* = 10 fetuses from 5 mothers), E17.5 (*n* = 10 fetuses from 6 mothers), and E18.5 (*n* = 11 fetuses from 6 mothers). ECG signals were measured in pregnant mothers and fetuses at each fetal stage.

In our analysis of heart rate and autonomic nervous system development in fetal mice, one-way ANOVA revealed significant main effects of developmental stage on heart rate (*F*[3, 39] = 8.026, *P* < 0.001, *η*_*p*_^2^ = 0.382; Figure 1A) and HF (ln) (*F*[3, 39] = 5.959, *P* = 0.002, *η*_*p*_^2^ = 0.314; Figure 1D); however, there were no significant main effects of developmental stage on LF (ln) (*F*[3, 39] = 1.193, *P* = 0.325, *η*_*p*_^2^ = 0.084; Figure 1C) or LF (ln)/HF (ln) (*F*[3, 39] = 0.908, *P* = 0.446, *η*_*p*_^2^ = 0.065; Figure 1E). Although not statistically significant, there was a tendency for developmental stage to affect STV (*F*[3, 39] = 2.756, *P* = 0.055, *η*_*p*_^2^ = 0.175; Figure 1B). *Post hoc* comparisons showed that fetal heart rate at E18.5 was significantly higher than that at E13.5, E15.5, and E17.5 (*P* < 0.001, *P* = 0.008, and *P* = 0.002, respectively) (Figure 1A). *Post hoc* analysis of HF (ln) indicated that HF (ln) at E18.5 and E17.5 was significantly lower than that at E13.5 (*P* = 0.003 and *P* = 0.028, respectively; Figure 1D). In summary, these results suggest that as fetal development progresses, parasympathetic activity (determined by HRV) decreases and heart rate increases.

**FIGURE 2:**
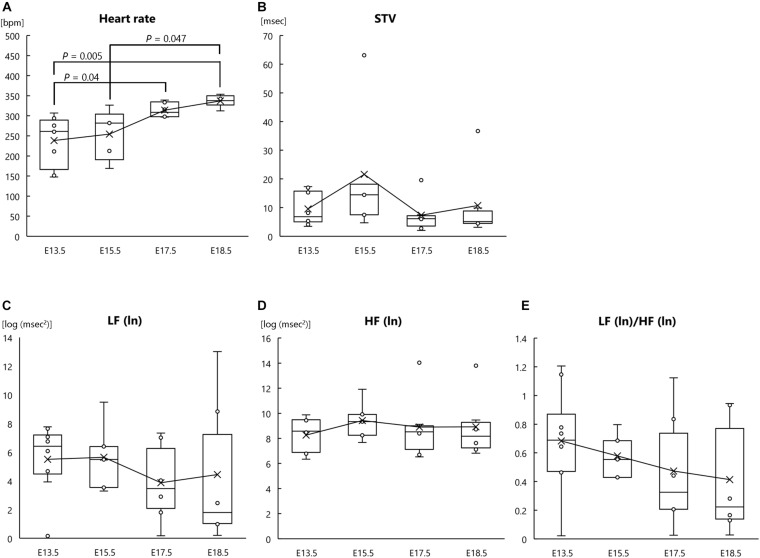
Heart rate and autonomic nervous activities at each gestational age in pregnant mother mice. **(A)** Heart rate, **(B)** short-term variability (STV), **(C)** natural logarithm of the low-frequency of the power spectrum [LF (ln)], **(D)** natural logarithm of the high-frequency of the power spectrum [HF (ln)], and **(E)** LF (ln)/HF (ln) ratio at the following gestational ages: E13.5 (*n* = 8), E15.5 (*n* = 5), E17.5 (*n* = 6), and E18.5 (*n* = 6). ECG signals were measured in pregnant mothers and fetuses at each gestational age.

In addition to analysis of fetal heart rate and HRV, we analyzed the heart rate and autonomic nervous functions of the mother mice. One-way ANOVA revealed a significant main effect of gestational age on heart rate (*F*[3, 21] = 6.672, *P* = 0.002, *η*_*p*_^2^ = 0.354; Figure 2A); however, there were no significant main effects of gestational age on STV (*F*[3, 21] = 1.271, *P* = 0.310, *η*_*p*_^2^ = 0.154; Figure 2B), LF (ln) (*F*[3, 21] = 0.372, *P* = 0.774, *η*_*p*_^2^ = 0.051; Figure 2C), HF (ln) (*F*[3, 21] = 0.320, *P* = 0.811, *η*_*p*_^2^ = 0.044; Figure 2D), or LF (ln)/HF (ln) (*F*[3, 21] = 0.709, *P* = 0.557, *η*_*p*_^2^ = 0.092; Figure 2E). *Post hoc* comparisons showed that maternal heart rate at E18.5 was significantly higher than that at E13.5 and E15.5 (*P* = 0.005 and *P* = 0.047, respectively) and that maternal heart rate at E17.5 was also significantly higher than that at E13.5 (*P* = 0.040) (Figure 2A). These results indicate that as parturition approached, the heart rate of mother mice increased.

## Discussion

In the present study, the heart rate of normal mouse fetuses was significantly increased at E18.5 compared with that at E13.5, E15.5, and E17.5. Thus, fetal heart rate seems to increase only immediately prior to birth, and mouse pups are usually born on E19.5. In our previous study, we also showed that fetal heart rate increased at E18.5 compared with that at E15.5 in both saline- and valproic acid-treated mouse fetuses (Kasahara et al., 2020). Interestingly, as shown by one-way ANOVA, LF (ln) and LF (ln)/HF (ln) ratio in mouse fetuses did not significantly change with developmental stage in the present study; therefore, sympathetic nervous activity remained unchanged during the fetal period, at least from E13.5 to E18.5. In contrast, fetal HF (ln), representing parasympathetic nervous activity, was significantly reduced at E17.5 and E18.5 compared with that at E13.5. Fetal STV, another indicator of parasympathetic function, also showed a tendency to reduce with developmental stage. Therefore, our results suggest that parasympathetic activity rather than sympathetic activity affects fetal heart rate and that a decrease in fetal parasympathetic activity toward the end of pregnancy causes the observed increase in the heart rate of fetuses. In our previous study, although LF (ln) and LF (ln)/HF (ln) decreased over the fetal period in fetuses treated with valproic acid, heart rate of these fetuses did not differ from that in the control group (Kasahara et al., 2020). Similar to the present findings, this previous result supports the idea that heart rate is regulated by the parasympathetic nervous system rather than the sympathetic nervous system.

It has previously been reported that parasympathetic cardiac innervation precedes sympathetic cardiac innervation in the fetal period (Vegh et al., 2016); although NCC-derived parasympathetic neurofilaments are present at the venous pole of the mouse embryo heart at E12.5, sympathetic nerves moving along the coronary veins reach the apex at the dorsal side of the heart at E15.5 (Hildreth et al., 2008; Nam et al., 2013; Vegh et al., 2016). It has also been reported that HRV is reduced by parasympathetic blockade alone but not by β blockade alone in chronically catheterized fetal lambs (Dalton et al., 1983). Thus, parasympathetic activity but not sympathetic activity at the fetal stage appears to generally affect fetal heart rate. Because fetal parasympathetic nervous function develops earlier than fetal sympathetic nervous function (Vegh et al., 2016), the heart rate could be more strongly influenced by the fetal parasympathetic nervous system during the late fetal period. Moreover, the increase in fetal heart rate immediately before birth could be a physiological response to the stress of parturition. The decrease in fetal parasympathetic activity during late pregnancy can alter the balance between the sympathetic and parasympathetic nervous systems; the relative dominance of the sympathetic nervous system at this stage might help prepare the fetus for the stress of birth. Indeed, it was previously reported that the release of cortisol and catecholamines increases markedly along with heart rate during labor in calves (Nagel et al., 2016; Nagel et al., 2019). In both equine and bovine neonates, a marked increase in heart rate and a decrease in HRV were observed in the immediate postnatal period, suggesting that sympathetic and parasympathetic activity were high and low, respectively (Nagel et al., 2019). Additionally, a study on sheep showed that neonatal blood noradrenaline levels increased 1 h after parturition (Segar et al., 1994). These observations strongly support our results, i.e., that an increase in heart rate and a decrease in parasympathetic activity occur just before birth at E18.5, indicating that the sympathetic nervous system is relatively dominant around birth.

In this study, the heart rate of mother mice increased significantly as they approached parturition. It is well known that heart rate increases as gestational age progresses in humans (Soma-Pillay et al., 2016; Loerup et al., 2019); thus, our results in mice are consistent with findings in humans. However, the autonomic nervous system activity of mother mice, as analyzed by HRV, did not change as gestational age progressed. The discrepancy between these heart rate and autonomic nervous activity results could have arisen due to the influence of humoral factors such as hormones. Indeed, infusion of the uterine contraction hormones prostaglandin F2α and prostaglandin E2 has been shown to increase heart rate in pregnant anesthetized women (Secher et al., 1982).

Our previous studies revealed that STV was reduced in the fetuses of FGR mice (Minato et al., 2018) and that LF (ln) and LF (ln)/HF (ln) ratio were reduced in the fetuses of ASD mice (Kasahara et al., 2020). These results indicate that the environment and disease strongly influence sympathetic and/or parasympathetic nerve function and development during the fetal period. The mechanism underlying these differences, however, remains to be elucidated. Because FGR mice are more likely to present with cerebral hemorrhage (Minato et al., 2018) and mice treated prenatally with valproic acid exhibit ASD-like behavior when they have matured (Kasahara et al., 2020), it is possible that an imbalance between sympathetic and parasympathetic nerve activity in the fetus affects disturbances in brain development and/or maturation. Considering these results, the increase in fetal heart rate and decrease in parasympathetic nervous activity immediately before birth might also play an important role in brain development and/or maturation. Although the present study focused on heart rate and sympathetic and parasympathetic nerve functions in healthy fetuses, future studies should aim to make similar findings in various pathological models to elucidate some of the pathological mechanisms of DOHaD-related diseases.

A limitation of our study was the anesthesia regimen: pregnant mice were anesthetized with a mixture of ketamine and xylazine, and isoflurane was used to maintain anesthesia. As ketamine is known to pass through the placental barrier and affect rodent fetuses (Zhao et al., 2014; Li et al., 2018), the anesthesia regimen may have been a factor that affected the results. Indeed, there was relatively large variation in the data in this study; it is possible that the anesthesia regimen affected this data variability. In addition, some studies have shown differences between pregnant and non-pregnant patients in sensory and motor block produced by anesthetics (Hirabayashi et al., 1996; Zhan et al., 2011); thus, sensitivity to anesthetics could potentially differ at each stage of pregnancy, and this may also have affected our results. However, while in mother mice we observed no change in autonomic nerve activity according to gestational age, in fetal mice we found that parasympathetic nerve activity decreased as developmental stage progressed. These trends suggest that the decrease in parasympathetic nerve activity observed in fetuses is a fetus-specific change, even if there was an effect of anesthesia.

In conclusion, we showed that fetal heart rate increases immediately prior to birth in normal mouse fetuses and that this change could be influenced by a decrease in fetal parasympathetic nerve activity. Our self-developed technique for measuring fetal ECG will be extremely useful for elucidating the physiological characteristics of fetuses in animal models of disease and in healthy animals.

## Data Availability Statement

The original contributions presented in the study are included in the article/supplementary material, further inquiries can be directed to the corresponding author/s.

## Ethics Statement

The animal study was reviewed and approved by the Committee on Animal Experiments in Tohoku University.

## Author Contributions

YKa and CY designed this study. YKa and CY performed the experiments. CY analyzed the data. YKa checked the data. YKa and CY wrote the manuscript. YKa, CY, MS, and YKi edited the manuscript. All authors contributed to the article and approved the submitted version.

## Conflict of Interest

YKa received funding from Shiguredo Inc., and declares that there are no employment, consultancy, patents, products in development, marketed products, or related interests, and there are no restrictions on the sharing of data and/or materials. The remaining authors declare that the research was conducted in the absence of any commercial or financial relationships that could be construed as a potential conflict of interest.
